# YAP/TAZ, beta-catenin, and TGFb pathway activation in medical plasma-induced wound healing in diabetic mice

**DOI:** 10.1016/j.jare.2024.07.004

**Published:** 2024-07-08

**Authors:** Anke Schmidt, Thomas von Woedtke, Klaus-Dieter Weltmann, Sander Bekeschus

**Affiliations:** a ZIK plasmatis, Leibniz-Institute for Plasma Science and Technology (INP), a member of the Leibniz Health Technologies Research Alliance, Felix-Hausdorff-Str. 2, 17489 Greifswald, Germany; b Institute for Hygiene and Environmental Medicine, Greifswald University Medical Center, Sauerbruchstr., 17475 Greifswald, Germany; c Department of Clinic and Policlinic for Dermatology and Venerology, Rostock University Medical Center, Strempelstr. 13, 18057 Rostock, Germany

**Keywords:** CAP, Cytokines, Diabetes mellitus, Differentially expressed genes, Reactive oxygen and nitrogen species, Redox processes

## Abstract

**Introduction:**

Hippo is a signaling pathway that is evolutionarily conserved and plays critical roles in wound healing and tissue regeneration. Disruption of the transcriptional activity of both Hippo-associated factors, the yes-associated protein (YAP), and the transcriptional co-activator with PDZ binding motif (TAZ) has been associated with cardiovascular diseases, fibrosis, and cancer. This makes the Hippo pathway an appealing target for therapeutic interventions.

**Objectives:**

Prior research has indicated that medical gas plasma promotes wound healing by delivering a combination of reactive species directly to the affected areas. However, the involvement of YAP/TAZ and other signaling pathways in diabetic wound healing remains unexplored.

**Methods:**

To this extent, ear wounds were generated and treated with gas plasma in streptozotocin (STZ)-induced diabetic mice. Transcriptome profiling at two wound healing stages (days 9 and 20 post-wounding) was performed in female and male mice. Additionally, we employed gene and protein expression analyses, utilizing immunohistological and −chemical staining of various targets as well as quantitative PCR and Western blot analysis.

**Results:**

Gas plasma treatment accelerated healing by increasing re-epithelialization and modifying extracellular matrix components. Transcriptomic profiling charting the major alterations in gene expression following plasma treatment was followed by a validation of several targets using transcriptional and translational quantification as well as localization analyses.

**Conclusion:**

Our study evaluated the cellular regulation of essential targets of the Hippo and related pathways such as YAP/TAZ, β-catenin, tumor growth factor β, and oxidative stress signaling after plasma treatment. The activation of genes, pathways, and their regulators is an attractive therapeutic aim for a therapeutic intervention in dermal skin repair in diabetic diseases using medical gas plasmas.

## Highlights

• Medical gas plasma promotes diabetic wound healing *in vivo*.
• Transcriptomic profiling identified the importance of the Hippo and related YAP/TAZ pathway.
• Treatment was concomitant with the translational upregulation of key antioxidant proteins.

## Graphical abstract

**Schematic of Hippo signaling pathway during wound healing in a diabetic mouse model**. The gas plasma-generated reactive species act as second messengers and stimulate several Hippo signaling targets (*e.g.*, upstream kinases, YAP, TAZ, downstream targets), ultimately promoting wound healing.

**Figure:**
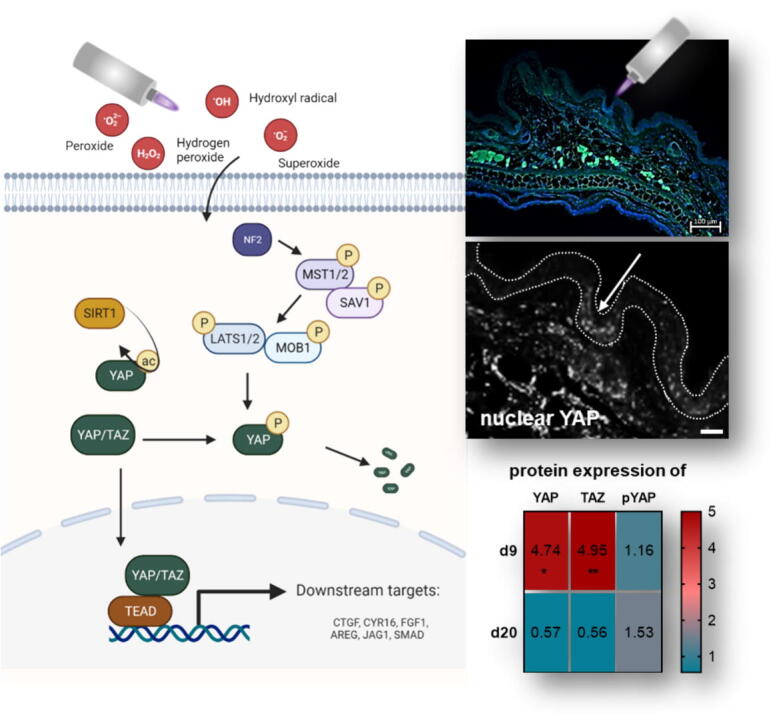

## Introduction

The skin, the body's largest organ, serves as both a structural and biological barrier to and first line of defense against environmental stressors (*e.g.*, microorganisms, chemicals, and physical trauma). The skin barrier comprises epidermal and dermal layers of cells, including the *Stratum corneum* (SC), the outermost and least permeable layer of the epidermis [1]. Besides its physical barrier function, the skin also contains immune cells, such as Langerhans cells recognize and respond to foreign substances and microbes, which helps to protect the body from infection. Various factors, including genetic disorders and environmental factors such as exposure to UV radiation, pollution, harsh chemicals, and mechanical wounding, can compromise the skin's barrier function. Injuries compromise the structural integrity and functionality of the skin. Normal wounds heal in well-organized and coordinated processes of homeostasis, inflammation, proliferation, and remodeling [2]. Understanding such complexities, signaling pathways, and therapeutic options that regulate skin repair is a primary aim of regenerative medicine.

Age and widespread diseases such as diabetes mellitus often result in pathological systemic changes and defective wound healing. According to the International Diabetes Federation, approximately 7.4 million people in Germany live with diabetes in 2021, representing 8.7 % of the population [3]. Diabetes mellitus is frequently correlating with lifestyle factors such as obesity and physical inactivity. In Germany, the majority of diabetes cases are attributed to type 2 diabetes [4]. Type 1 diabetes commonly manifests in childhood or early adulthood, constituting around 5–10 % of total diabetes cases [5]. Persistent wounds in individuals with diabetes predominantly include venous leg ulcers, diabetic foot ulcers, and pressure ulcers [6]. Several host-related factors hinder the healing process, including neuropathy, elevated blood glucose levels, and compromised immune responses [7]. Additionally, reactive oxygen and nitrogen species (summarized as ROS) play a role in the pathogenesis of diabetes by promoting insulin resistance, β-cell dysfunction, inflammation, vascular complications, and oxidative damage [8]. Slow wound healing further increases the risks of infection. Various rodent models, such as the streptozotocin (STZ) models, have been established to investigate delayed wound healing in diabetes [9], [10]. As a result of the targeted damage or loss of pancreatic islet β-cells, the mice experience human type 1 diabetes characteristics with polydipsia, polyuria, hyperglycemia, and insulin deficiency [11].

In skin wound repair, Hippo pathway signaling is central for a cascade of kinases that phosphorylate the effectors of transcriptional co-activator with PDZ-binding motif (TAZ) and yes-associated protein (YAP) [12]. YAP/TAZ have emerged as crucial contributors to tissue regeneration, playing pivotal roles in various cellular processes such as angiogenesis, differentiation, proliferation, migration, survival, apoptosis, and by regulating vascular sprouting and barrier formation [13], [14]. This implies that YAP and TAZ could be potential therapeutic targets in cases of impaired wound healing. Upon activation of the Hippo pathway (“on”), YAP and TAZ undergo phosphorylation and inhibition by upstream kinases. Conversely, when the Hippo pathway is deactivated, YAP and TAZ undergo dephosphorylation and translocate to the nucleus (“off”). There, they bind to transcription factors and co-activators, facilitating gene expression and their regulation [15]. YAP and TAZ activation also corresponds to cell adhesion and mechanical signals cells receive from tissue architectures and surrounding extracellular matrix (ECM) [16].

Treatment strategies targeting defective wound healing include wound debridement, negative pressure wound therapy, nutritional support, medications, surgery, dressing, and topical agents [17]. However, the optimal treatment strategy for diabetes will depend on the individual's specific needs and circumstances. Moreover, the concept of redox control was introduced in wound healing two decades ago [18]. Redox processes comprise the electrons transfer between molecules upon ROS exposure subsequent to oxidative eustress, activating corresponding pathways [19]. The gas plasma technique has been investigated for its potential in wound healing by targeting cellular redox signaling (reviewed in [20], [21], [22], [23], [24], [25]). This novel technology involves a partially ionized gas containing ROS, ions, electrons, and excited atoms or molecules [26]. Consequently, gas plasma modulates a multitude of cellular processes associated with redox pathways. It has the potential to be beneficial in targeting numerous specific pathways related to wound healing [27] together with antimicrobial [28], [29] and antiviral effects [30], [31], [32]. While several preclinical studies in rodent models were successfully applied [33], [34], [35], [36], also clinical plasma therapy showed beneficial effects in patients with ulcers and other non-healing wounds [37], [38], [39].

Despite the treatment success with cold atmospheric-pressure plasma devices for wound healing applications, there is limited direct evidence of improved diabetic wound healing that elucidated the molecular mechanisms. In this work, we identified a central role of YAP/TAZ signaling in STZ-induced diabetic mice with artificial ear wounds following exposure to gas plasma-derived ROS. In addition, we identified several targets that concomitantly were regulated in gas plasma-accelerated wound healing, including β-catenin tumor growth factor beta (TGFβ) and oxidative stress (Nrf2) signaling as well as chemokine and cytokine release.

## Material and methods

### Ethics statement and wounding

SKH1-hr hairless immunocompetent mice (Charles River Laboratories, Germany) within the age range of 8–10 weeks were housed with a 12-hour light–dark cycle and had free access to food and water. The mice were employed for wound healing studies following protocols approved by the local ethics committee (approval code: 7221.3–1-044/16, Greifswald University Medical Center, Germany), adhering to the guidelines for the use and care of animals in the laboratory and the NIH rules for Use and Care of Animals in the Laboratory. Streptozotocin (STZ, 4 mg/mL in 50 mM sodium citrate buffer) was administered intraperitoneally (i.p., 50 mg/kg) five times over 120 h [40]. During STZ-treatment mice received 10 % sucrose in regular water to maintain hyperglycemia and free access to normal food chow [11]. On the experimental day of wounding (d0, 20 days after diabetes induction), blood glucose levels were monitored under ketamine (1.9 mg/mouse) and xylazine (0.19 mg/mouse) sedation, with criteria for diabetes being glucose levels > 8.3 nmol/L [41]. Full-thickness dermal wounds in the range of 2.5–4 mm^2^ were set on both ears by a microscissor as previously described [42]. Wound tissues were collected after animal sacrifice either on day 9 or day 20 post-injury. The isolation of primary skin cells was conducted using enzyme-mediated removal and digestion of the epidermis and dermis, following the instructions of the epidermis dissociation kit, utilizing gentleMACS C tubes and gentleMACS technology (OctaMACS). Afterward, primary cells were sieved using SmartStrainers (70 µm, Miltenyi Biotec, Germany) to obtain viable single-cell suspensions and cultured for 10 days in EMEM medium (PromoCell, Germany) containing 10 % fetal bovine serum, 1 % L-glutamine, and penicillin/streptomycin (Sigma-Aldrich, Germany) at 37 °C and 5 % CO_2_.

### Exposure to medical gas plasma and re-epithelialization measurements

**Fig. 1:**
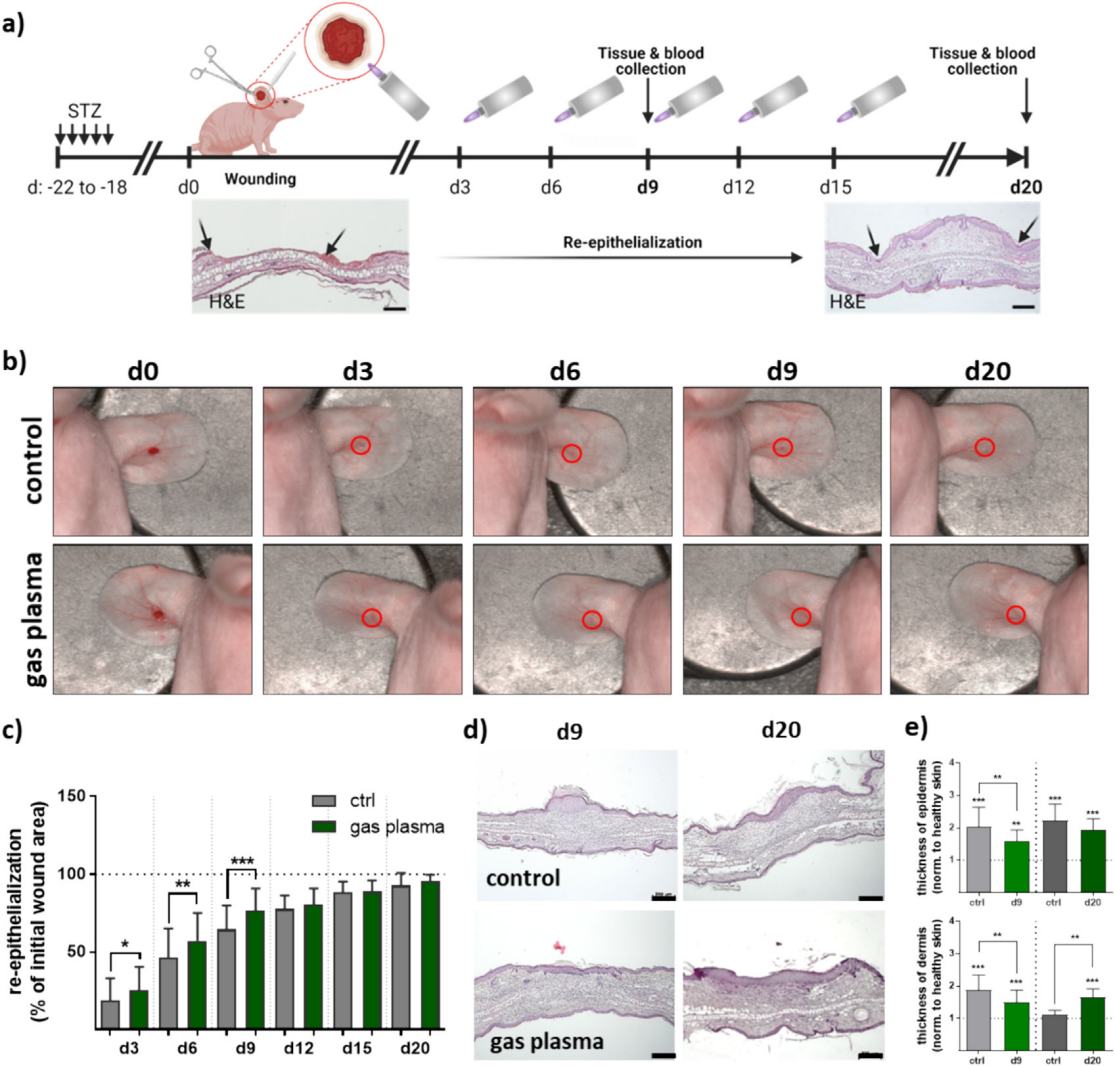
**Repeated gas plasma treatment shortened wound closure in diabetic mice.** (**a**) Schematic timeline of gas plasma treatment in diabetic mice illustrates wound closure and treatment methods. STZ was administered i.p. on five consecutive days. After 18 days (corresponding to d0), ear wounds were generated. Representative images of hematoxylin and eosin (H&E) staining after wounding illustrate the failure of the upper epidermal and dermal layer on d0 (left) and re-epithelialization with migration of skin cells into the wound bed on d20 (right). Wound tissue and blood were collected on d9 or d20, respectively. (**b**) Representative images of wound healing on days 0, 3, 6, 9, and 20 in untreated control (ctrl) mice (upper panel) and gas plasma-treated mice (circle: wound and treatment area). (**c**) The wound closure rate (epithelialization) was plotted as percentage of original wound area reduction over time for treated mice when compared to the untreated controls (n ≥ 4). (**d**) Representative H&E staining of ear wounds on days 9 and 20 showing re-epithelialization stages and histological alterations after gas plasma treatment (10 s). (**e**) Thickness quantification of epidermis (upper diagram) and dermis (lower diagram) at both endpoints in gas plasma-treated and untreated wounds of mice compared to healthy (unwounded) skin. Data are presented mean ± S.D.; *p < 0.05, **p < 0.01, and ***p < 0.001 compared to controls; scale bars are 200  µm.

Medical gas plasma treatment was done using an argon plasma jet kINPen MED (neoplas med GmbH, former neoplas tools GmbH, Germany), as outlined before [42]. Briefly, wounds were subjected to gas plasma treatment utilizing the tip of the plasma jet effluent, maintained at a consistent distance of 8 mm with the aid of an autoclavable spacer. Treatment of ear wounds lasted for 10 s every three days starting on the day of wounding (d0), while control wounds remained untreated (ctrl). The experiments were conducted over 9 days (four gas plasma treatment sessions) or 20 days (six gas plasma treatment sessions) (Table1). Re-epithelialization and various aspects of the wound healing process were analyzed on the day of setting a dermal ear wound (d0) and on every third day after wounding. The study regime is shown for the treatment groups and endpoints investigated (Fig. 1**a**). Skin cells (mix of keratinocytes, fibroblasts, and immune cells) were isolated from the wound of control and plasma-treated animals after both 9 days and 20 days [42], and examined in immunofluorescence analyses.

**Table 1:** Overview of experimental groups, time of sacrifice after wounding, treatment methods, and gender (d, day; s, seconds).

**group**	**endpoints**	**treatments**	**quantity (♂)**	**quantity (♀)**
ctrl	d9	0 s	8	8
gas plasma		every 3rd day over 9 day (4x), 10 s	8	8
ctrl	d20	0 s	10	8
gas plasma		every 3rd day over 20 days (6x), 10 s	10	8

### RNA and protein extraction from homogenized ear tissue

After harvesting wound tissues from one ear on both endpoints (d9 and d20), fresh samples were snap-frozen in liquid nitrogen and, for subsequent analysis, stored at −80 °C. To assess the expression levels of large numbers of genes simultaneously, transcriptome and gene expression validation experiments were carried out after tissue homogenization in RNA lysis buffer (Bio&Sell, Germany) using a FastPrep-24 5G homogenizer (MP biomedicals, Germany). For protein expression, samples were lysed in RIPA buffer supplemented with protease and phosphatase inhibitors (cOmplete Mini, phosSTOP, PMSF; Sigma-Aldrich, Germany).

### Transcriptome microarray analysis

After extraction of total RNA using an RNA Miniprep kit (Bio&Sell, Germany), integrity and purity of the input RNA were quality-controlled using the Bioanalyzer 2100 (Agilent, USA; n ≥ 4). RNA from two independent experiments was combined and labeled with the fluorescent dye Cy3 using Low Input Quick Amp One-Color Labeling Kit (Agilent, USA). After purification and quantification of cRNA, samples were blocked and hybridized onto SurePrint G3 custom GE 8 × 60 K chips (OakLabs, Germany) for 17 h at 65° C in a hybridization oven (Agilent, USA) following previously established procedures [43]. After extensive washing steps in several wash buffers, fluorescence signals were measured using an Agilent SureScan microarray scanner. Using Agilent Feature Extraction Software, signal intensities were analyzed and normalized, and downstream analysis of Agilent microarray data, such as statistical comparison and hierarchical clustering, was done using Genespring software (Agilent, Germany).

### Differentially expressed genes (DEG) and data analysis

Results were organized based on the treatment regime into two groups: one treated with a 10-second gas plasma treatment and the other untreated (control group). Statistical analysis on grouped data was performed using multiple testing corrections: Differentially expressed genes (DEG) were identified based on statistical significance with an adjusted p-value below a threshold of 0.05. Additional criteria for considering a gene as differentially expressed included a fold change (FC) of ≥ 2.0 or ≤ 0.5. Functional clustering of sets of co-regulated genes was done. Down-stream analysis included categorization based on protein classes, molecular functions, and examination of upstream and downstream targets of the genes using PANTHER software pathway analysis. Afterward, genetic networks were created to visualize and understand functional relationships among genes. The created genetic networks are based on known associations in relevant databases, providing insights into the interplay of genes within biological processes.

### Quantitative polymerase chain reaction (PCR)

Quantitative PCR (qPCR) was used to determine mRNA levels. Briefly, 1  μg of RNA was transcribed into cDNA, and gene-specific primers (BioTez, Germany) were used for the amplification of specific targets by qPCR using SYBR Green I Master (Roche Diagnostics, Switzerland). Housekeeping genes *GAPDH* and *RPL13A* (Table S1) were chosen as internal controls to normalize the gene expression data. These reference genes were selected because their expression levels were not affected by gas plasma exposure, ensuring a stable reference for normalization. Expression levels were identified using the ΔΔCT method, which involves comparing the cycle threshold (CT) values of the gene of interest with those of the reference genes.

### Protein analyses using the WES system

Protein targets were validated based on their significance within the main cellular responses. These included molecules of the Hippo (*e.g.*, YAP, TAZ, pYAP/TAZ) and Nrf2-pathway (*e.g.*, nuclear Nrf2, HO-1) as well as antioxidative response targets (Cat, Sod1). Gapdh served as a reference protein. Western blot analysis) was performed using the WES system (all antibodies from Cell Signaling Technology, Germany, as described in the protocols and guidelines provided by the manufacturers (ProteinSimple, Germany). Compass Software (ProteinSimple, Germany) was used to analyze and quantify band intensities obtained from an experimental setup. A comparative analysis was performed where the intensity of each band is measured relative to the corresponding control.

### Histology, immunohistochemistry, and immunofluorescence analysis

Wound regions of the other ears were collected and fixed overnight in 4 % paraformaldehyde (Sigma-Aldrich, Germany) on days 9 and 20 post-wounding. Tissues were initially embedded in paraffin and cut into 5  µm-thick sections using a microtome. Tissue sections were stained with both hematoxylin and eosin (H&E; Carl-Roth, Germany) or PSR (Direktrot 80; Sigma-Aldrich, Germany) as described [44] for visualization of collagen fibers. For immunohistochemistry, tissues were stained with YAP, TAZ, and pYAP (Ser127) primary antibodies, and staining was visualized using SignalStain boost IHC detection reagents (all Cell Signaling Technology, Germany). For immunofluorescence microscopy analysis of several proteins, fixed skin cells (retrieved from untreated or *in vivo* gas plasma-treated skin) and tissue samples were washed and permeabilized with Triton X-100 (0.01 % in PBS; Sigma-Aldrich, Germany). After incubation with primary antibodies targeting β-catenin, E-cadherin, collagen I, Nrf2, iNOS, and TGFβ1 (all Cell Signaling Technology, Germany), cells or wound tissues were incubated with Alexa Fluor 488 or 594 conjugated secondary antibodies (Life Technologies, Germany) and DAPI to visualize nuclei. Mounted sections (VectaShield; Biozol, Germany) were monitored in fluorescence microscopy using an Axio Observer Z.1 (Zeiss, Germany).

### Statistical analysis

Experiments involving tissues were conducted with at least four animals per group. All *in vitro* experiments were repeated three times. Mean values, along with standard deviations (SD), were reported unless stated otherwise. Significance levels were indicated using asterisks by *p < 0.05, **p < 0.01, or ***p < 0.001. Statistical and graphical analysis was performed with *prism* 7.04 (GraphPad Software, USA). Unpaired *Student's t*-test was used for comparisons between two groups. One-way analysis of variances (ANOVA) was used for three or more group comparisons.

## Results

### Gas plasma treatment promoted wound healing in diabetic SKH1 mice

To induce diabetes mellitus in immunocompetent nude SKH1 mice, the diabetogenic substance streptozotocin (STZ) was injected daily on five consecutive days. The medical gas plasma's potential to promote healing was investigated in dermal full-thickness ear wounds in this diabetes type 1 model. Thus, murine ear wounds either received gas plasma treatment (10 s) every third day over 9 (d9) and 20 (d20) days after wounding or were left untreated (ctrl). Arrowheads indicated the hyperproliferative epidermis at the wound edge, indicating the migrating epidermal front (Fig. 1**a**). Wound closure (red circle = wound area) was shown in representative images in gas plasma-treated and control mice (Fig. 1**b**). The healing rate showed improved re-epithelialization in the gas plasma–treated compared to the untreated wounds, which was comparable in both females and males and therefore summarized. Quantitative analysis for each treatment day showed significantly accelerated wound closure beginning with days 3, 6, and 9 compared to untreated controls (ctrl). A near-complete re-epithelialization (>95 %) was achieved on day 20 (Fig. 1**c**). To show that the skin dermis defects were gradually closed by newly formed granulation tissue that was covered with epidermis, we employed histological observation by hematoxylin and eosin (H&E) staining during the healing process (Fig. 1**d**). We quantified differences between the experimental groups in terms of thickness of epidermal and dermal compartments such as granulation tissue at the center and margin zone in the defect areas. As expected, mice treated with gas plasma exhibited a significantly higher proportion of re-epithelialized wounds and granulation tissue, along with a slightly lower epidermal thickness after injury compared to untreated controls (Fig. 1**e**, upper diagram). An increase in epidermal thickness and thickening of granulation tissue in the wound zone was identified with a statistically significant difference to healthy skin at both endpoints (Fig. 1**e,** lower diagram). On day 20 post-wounding, the thickness of the newly formed epidermal layer was still higher than in healthy skin but comparable in both experimental groups and without any observed scar formation. Moreover, inflammatory cell aggregation or multinucleated, swollen macrophages are common features of injury-induced inflammation. Scattered cell aggregation and alterations in immune cell morphology were not detected in H&E-stained tissues in all treatment groups, suggesting normal, physiological healing processes in all experimental groups.

### Gas plasma-induced wound healing altered gene expression patterns in diabetic mice

**Fig. 2:**
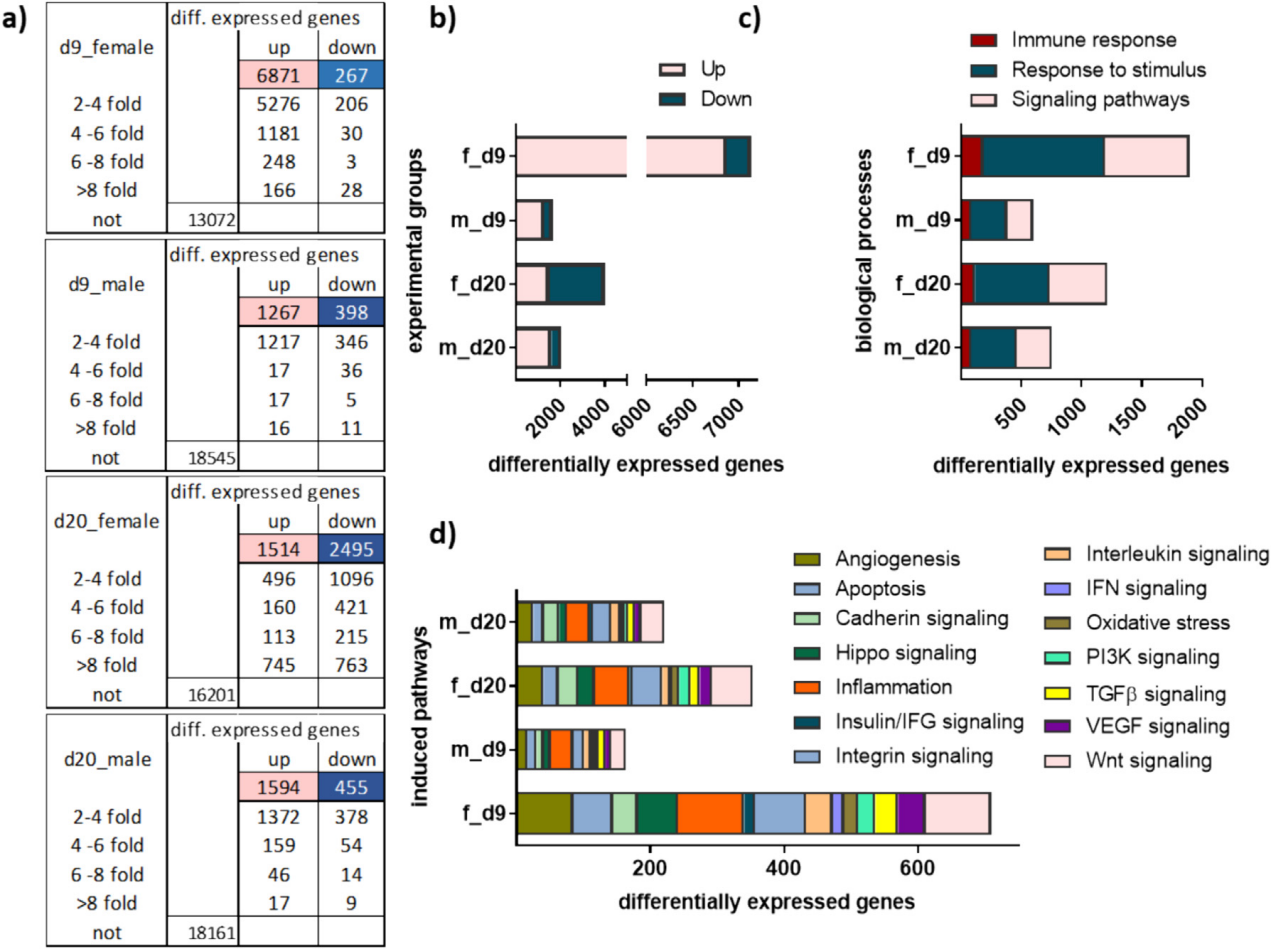
**Gas plasma treatment altered gene expression profiles in wounds of diabetic mice.** Wounds of SKH1-STZ mice were either exposed to gas plasma (10 s) or left untreated (ctrl). (**a**) Tables show significantly up- (pink) and downregulated (cyan) genes after four (d9) or six (d20) gas plasma treatment sessions of wounds compared to wounds of untreated control mice. (**b-d**) Diagrams showing differentially expressed genes (DEG) for all experimental groups at d9 and d20 in females and males: (**b**) number of up- and downregulated DEG, (**c**) top biological processes of significantly regulated genes including immune response, response to stimulus, and signaling pathways, and (**d**) top pathways. Males and females were used on d9 and d20 (n ≥ 4). (For interpretation of the references to color in this figure legend, the reader is referred to the web version of this article.)

With gender-specific differences and heterogeneity in mind [45], we investigated gas plasma-mediated effects in females and males separately at both endpoints (d9, n = 4 for both genders; d20, n = 4 for females, n = 5 for males) in a global transcriptomic approach. Groups were displayed as f_d9, m_d9, f_d20 and m_d20, respectively (f = female; m = male). Wound tissue transcripts of each treated group were subjected to functional gene expression analysis to evaluate differential gene expression compared with their untreated counterparts. Differentially expressed genes (DEG) were defined as such if having a false discovery rate (FDR) value (p-adjusted) of ≤ 0.05 and a fold change (FC) of ≥ 2 or ≤ 0.5. Compared to untreated mice, gene expression was significantly altered after four (d9) or six (d20) gas plasma treatment cycles in females and males, respectively. On d9, analysis of gas plasma-treated mice transcripts results in 7138 DEG, 96 % upregulated (6871) and 4 % downregulated (2 6 7) in comparison to untreated females (Fig. 2**a**). Considerably fewer DEG were found in males (1665, 76 % up- and 24 % downregulated). Similarly, fewer DEG were identified in gas plasma-treated males at later time points (d20) (2049, 78 % up- and 22 % downregulated). Comparison of the DEG of females on d20 to all others demonstrated a total of 4009 genes, 38 % induced and 62 % repressed. The number of up- and downregulated DEG following the different treatments are summarized in Fig. 2**b**, underlining the gender-specific differences and higher number of DEG in females on d9. The identified DEGs were further subjected to functional analyses by employing the PANTHER database. The diagram depicts several biological processes in all four treatment groups. The majority of these biological processes are related to respone to stimulus, signaling pathways, and immune responses (Fig. 2**c**). Next, those categories and pathways that are equally affected by gas plasma treatment were visualized, *e.g.*, angiogenesis, apoptosis, inflammation, oxidative stress responses, and several signaling pathways such as cadherin, fibroblast growth factor (FGF), insulin/insulin growth factor (IFG), integrin, interleukins (IL), interferon (IFN), phosphor-inositol 3 kinase (PI3K), tumor growth factor beta (TGF β1), vascular endothelial growth factor (VEGF), Hippo, and Wnt signaling (Fig. 2**d**).

### Gas plasma-treated wounds showed activated Hippo signaling pathway signatures

**Fig. 3:**
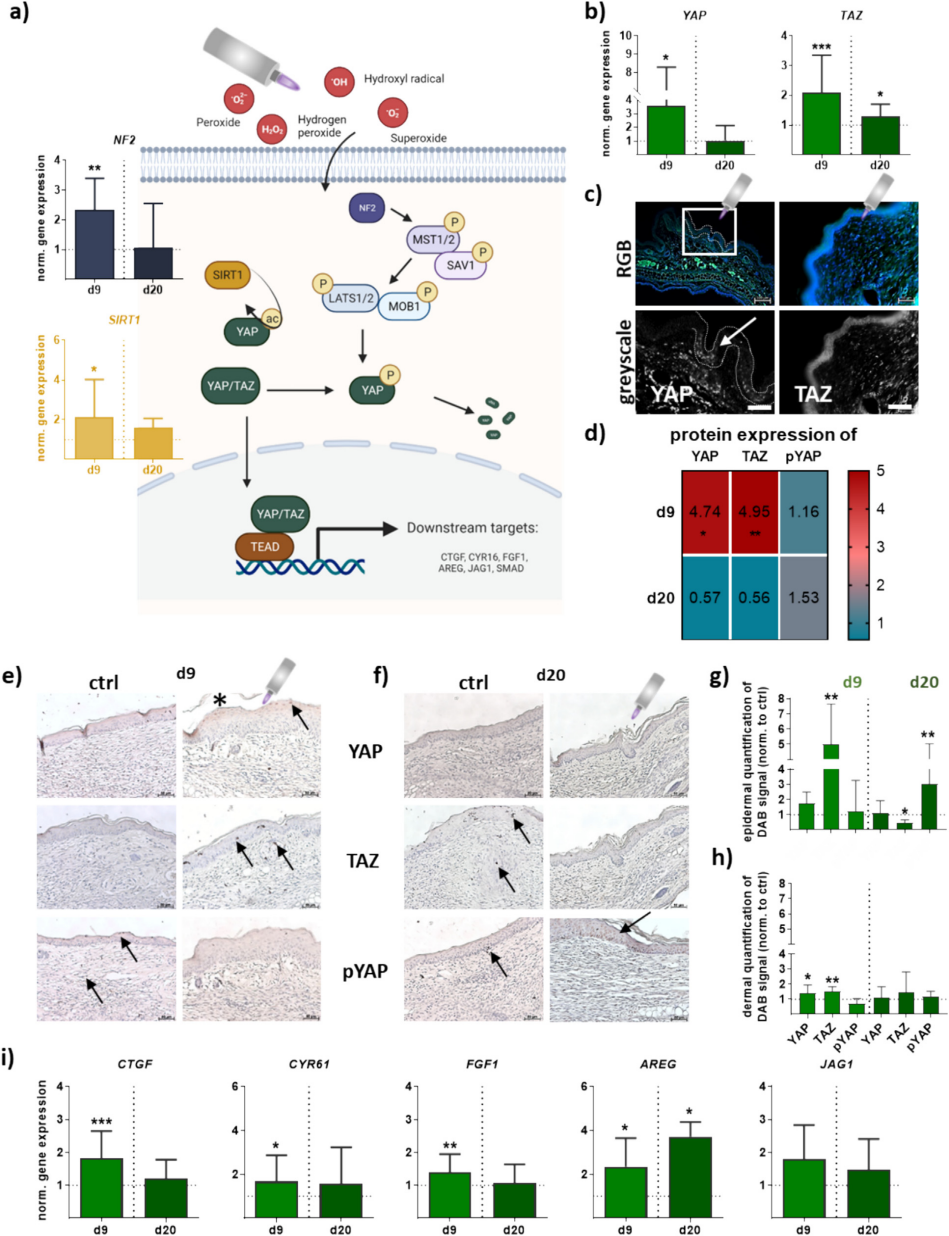
**Gas plasma modulated the Hippo signaling pathway in wounds of diabetic mice.** Ear wounds of mice were treated with gas plasma as described. (**a**) Schema of Hippo signaling including gene expression levels of upstream kinases (*e.g.*, *NE2*, *SIRT1*), and (**b**) the key factors *YAP1* and *TAZ* on both endpoints. (**c**) Tissue sections were antibody-stained for either YAP or TAZ to reveal their expression and subcellular localization. (**d**) Protein expression levels of YAP and TAZ and YAP phosphorylation (pYAP) were determined using WES analysis. (**e-f**) Wounds were stained for either YAP TAZ or pYAP on d9 (**e**) and d20 (**f**). Arrows indicate nuclear YAP/TAZ in basal layer cells; asterisk indicates flattened suprabasal cells with cytoplasmic and nuclear YAP. (**g-h**) Quantification of diaminobenzidine (DAB) signal of several targets (YAP, TAZ, pYAP) in epidermal (**g**) and dermal layers (**h**) on both endpoints. (**i**) mRNA expression levels of downstream targets (*e.g.*, *CTGF, CYR61, FGF1, AREG, JAG1*). Males and females were used on d9 and d20. Data are presented mean ± S.D.; *p < 0.05, **p < 0.01, ***p < 0.001 when compared to untreated controls (ctrl).

We next validated protein targets based on their significance within main cellular responses to wound healing. These included targets of the Hippo pathway, particularly YAP expression, which is one of the central components of tissue homeostasis and a key regulator of a magnitude of signaling pathways (schematic overview in Fig. 3). To elucidate the molecular basis of the relationship between YAP and TAZ, upstream and downstream target gene expression of “on” or “off” Hippo stages, we characterized the mRNA expression of major targets. Merlin (or neurofibromatosis type II, NF2), a membrane-cytoskeleton scaffolding protein, is an upstream regulator of the Hippo pathway [46]. NF2 mRNA expression was strongly induced upon gas plasma treatment on d9 but similar to untreated control expression on d20. Sirtuin 1 (SRT1), a nuclear enzyme that deacetylates transcription factors that contribute to cellular regulation and reaction to stressors [47], is downregulated in cells that have high insulin resistance, and inducing its expression increases insulin sensitivity, suggesting the molecule is associated with improving insulin sensitivity [48]. *SIRT1* expression was significantly increased in early wound healing stages compared to late stages, as shown by mRNA expression analysis (Fig. 3**a**). Next, we quantified the extent, expression, and subcellular localization of major targets of the core Hippo kinase cascade YAP and TAZ. Gas plasma treatment significantly increased the expression levels of YAP and TAZ mRNA in wounds (d9). At late time points (d20 after wounding), this increased expression of YAP and TAZ was mainly abrogated (Fig. 3**b**). We next investigated the localization of YAP and TAZ in wound tissue, confirming nuclear and cytoplasmic staining in the epidermis basal cell layer after plasma treatment (arrow, Fig. 3**c**). Quantitative western blot protein analysis revealed significantly increased YAP and TAZ levels in gas plasma-treated wounds on d9 but not d20 post-wounding (Fig. 3**d**), while YAP phosphorylation was not significantly affected. The YAP/TAZ-related serin-threonine kinases, mammalian sterile 20-related 1 and 2 kinases (*MST1/2*), large tumor suppressor 1 and 2 kinases (*LATS1/2*), and their upstream kinases, the MOB kinase activator 1A and 1B (*MOB1*), and Salvador 1 (*SAV1*), respectively, were not strongly affected on both experimental endpoints (Fig. S1**a**). We also evaluate whether YAP and TAZ contribute to skin repair after wounding and found, particularly in highly flattened squamous cells that were terminally differentiated, a strong YAP staining (DAB signal) in gas plasma-treated wounds (Fig. 3**e-f**, star sign). Nuclear staining in epidermal and dermal layers was visible for YAP and TAZ on d9. Phosphorylated YAP as an indicator for an activated Hippo signaling was visible at d20 (arrows, Fig. 3**e-f**). The quantification of YAP/TAZ and pYAP in the wound areas including epidermis (Fig. 3**g**) and dermis (Fig. 3**h**) 9 and 20 days after wounding validated findings suggesting that levels of both YAP and TAZ were elevated after wounding. pYAP was strongly expressed in the plasma-treated and untreated wound cells at d20 but not at d9. We further examined the transcriptional changes of various transcription factors regulated by YAP/TAZ, such as several well-characterized TEA domain family members in mammals (*TEAD1–TEAD4*) linked to most YAP/TAZ-directed biological functions [14]. We found a marked upregulation of TEADs in dependence on the family member and days after wounding (Fig. S1**b**). In addition, the interaction between TAZ and TEADs is essential for the matricellular proteins Cyr61 (cysteine-rich angiogenic inducer 61) and CTGF (connective-tissue growth factor) transcription in several cells [49], [50]. We, therefore, analyzed the expression of both corresponding integrin signaling regulators. *CTGF* and *Cyr61* expression increased significantly and peaked at d9 after wounding in contrast to untreated control tissue, indicating that the enhanced *CTGF* recruitment in gas plasma-treated wounds was indeed accompanied by the increased expression of YAP/TAZ signaling. Other downstream targets were differentially expressed as shown for fibroblast growth factor (*FGF1*), amphiregulin (*AREG*), an epidermal growth factor (EGF) family member, and jagged 1 (*JAG1*) (Fig. 3**i**).

### Gas plasma-treated wounds showed altered TGFβ and ECM signatures

**Fig. 4:**
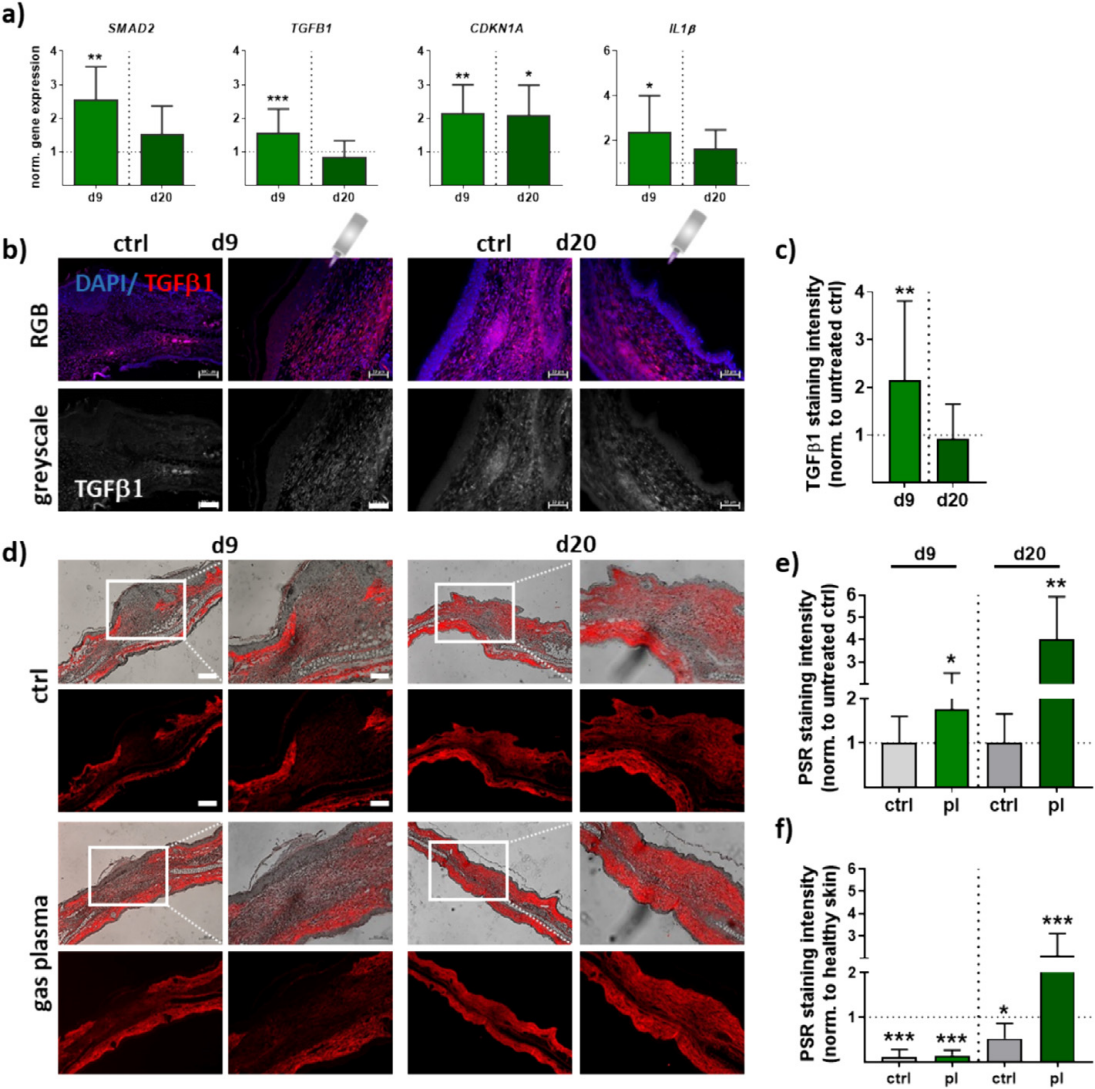
**Gas plasma-treated wounds showed a modulated gene expression and connective tissue stain dependent on wound healing stage.** Ear wounds of mice were treated with gas plasma as described. (**a**) Gene expression levels of transforming growth factor (*TGFβ*) signaling including *SMAD2, CDKN1A,* and interleukin 1 (*IL1β*) on both endpoints. (**b**) Immunofluorescence analysis of TGFβ1 and staining quantification on d9 and d20. (**c**) Quantification of TGFβ1 staining on both time points. (**d**) Representative picrosirius red (PSR)-stained connective ear tissue on days 9 (left) or 20 (right) in gas plasma-treated (lower panel) compared to untreated ear wounds of mice (upper panel) showing granulation tissue with collagen fibers (red) in brightfield (left images) and fluorescence microscopy (right images). Enlarged graphs display higher magnification. (**e-f**) Quantification of PSR using ImageJ software compared to untreated (**e**) and healthy (**f**) ear tissue showing fiber intensity on d9 and d20. Data are presented as mean ± S.D. *p < 0.05, **p < 0.01, ***p < 0.001 when compared to control groups (ctrl) with Students *t*-test (a, c) or ANOVA (e-f). Scale bars are 200 µm (d), 100 µm (d, higher magnifications), and 50 µm (b). (For interpretation of the references to color in this figure legend, the reader is referred to the web version of this article.)

YAP/TAZ are also regulated by other signaling pathways, including the transforming growth factor β1 (TGFβ1) pathway, which can modulate their activity and localization [51]. The expression level of *SMAD2*, a transcription factor that is activated by TGFβ signaling [52], remained higher than in controls in the wound area, suggesting that the TGFβ1 signal transduction depends on the activation of Smad2 and caused the rapid induction of p21 (gene name CDKN1A) transcriptional expression. This downstream target of TGFβ1 is a cyclin-dependent kinase inhibitor having a specific role in cell cycle regulation and cellular responses to stress [53]. We observed that *CDKN1A* expression was increased in the gas plasma-treated samples. Interleukin 1 beta (*IL1β*) was stimulated on d9 compared to controls but not after 20 days, suggesting a transient gas plasma effect (Fig. 4**a**). Using immunofluorescence microscopy, we next analyzed the expression pattern of TGFβ1 in control and gas plasma-treated wounds at different wound healing stages. TGFβ1-positive areas were noted in the dermal layers containing fibroblasts, both at the wound site and in adjacent tissues. During early wound healing stages at day 9, TGFβ1 expression was strong and much higher in gas plasma-treated compared to untreated wounds. On day 20, no clear difference was detected between the control and gas plasma-treated wounds (Fig. 4**b**). Quantification of TGFβ1 confirmed findings of an increased release of TGFβ1; its expression decreased to similar extents in the wound sites on d20 for all experimental groups (Fig. 4**c**). YAP/TAZ also respond to changes in extracellular matrix (ECM) rigidity and stiffness with an activation. To assess any potential effect of gas plasma exposure on wound cells and matrix deposition, we analyzed picrosirius red (PSR) staining to identify both thin and thick collagen fibers in wound regions. Representative PSR-stained connective ear tissues on days 9 (left) and 20 (right) in gas plasma-treated (lower panel) compared to untreated mice (upper panel) highlight the granulation tissue, including collagen fibers (red) in brightfield (left images) and fluorescence (right images) microscopy. Enlarged images displayed a higher density of collagen on d20 in gas plasma-treated wound tissue compared to untreated controls (Fig. 4**d**). The quantification of total collagen and type I and III collagen in PSR-stained slices were conducted and compared to untreated but wounded ear tissue showing a significant increase of fibers on d9 and 20 following gas plasma exposure (Fig. 4**e**). In contrast, PSR-stained fibers were significantly decreased on d9 when compared to healthy tissue but not for gas plasma-exposed mice on d20 suggesting a time-dependent gas plasma effect on ECM deposition (Fig. 4**f**).

### Gas plasma-treated wounds presented junctional and stress-related genes

**Fig. 5:**
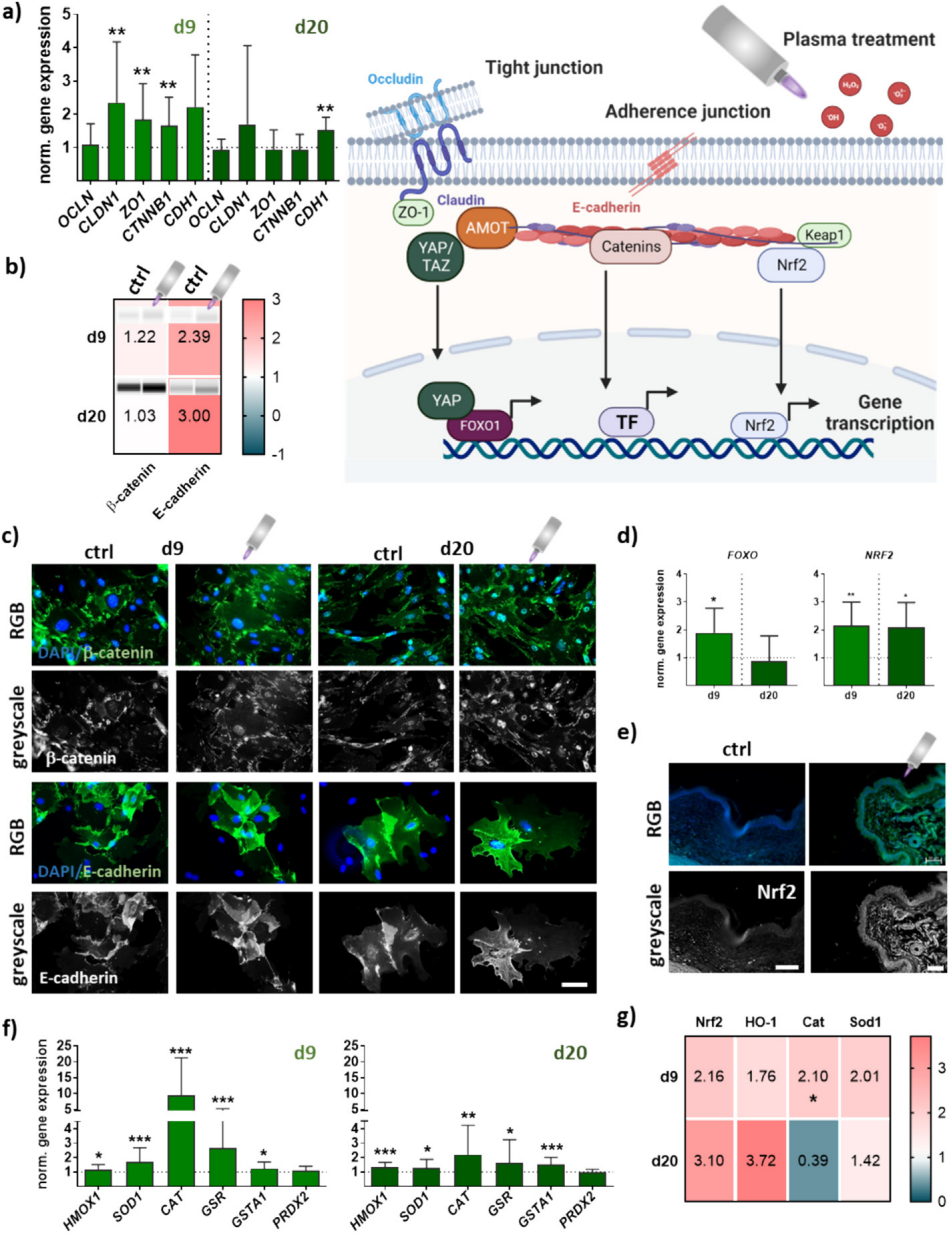
**Gas plasma wound exposure promoted expression of junctional and oxidative stress-induced targets**. Ear wounds of mice were treated with gas plasma as described. Overview of the connected pathways at junctional cell–cell connections and gas plasma-induced signaling. (**a**) Gene expression analysis of junctional targets of tight junctions (*e.g.*, *OCLN, CLDN1, ZO-1*) and adherence junctions (*e.g.*, *CTNNB1, CDH1*) was performed with total RNAs isolated from the wound regions on both endpoints (d9/d20). (**b**) Protein expression levels of β-catenin and E-cadherin were quantified using WES analysis on d9 (upper panel) and d20 (lower panel) compared to wound tissue from untreated mice. Males and females were used on d9 and d20. (**c**) Immunofluorescence analysis. Skin cells of untreated (left panel) and gas plasma-treated ear wounds of SHK1-STZ (right panel) mice were isolated, cultured, and stained for β-catenin (upper images) and E-cadherin (lower images) in *in vitro* gas plasma-treated (right images) compared to untreated (left images) skin cells. (**d**) Quantification of mRNA expression of transcription factors (*FoxO, NRF2*). (**e**) Distribution of Nrf2 in wound tissue after immunofluorescence staining on day 9. (**f**) qPCR-based expression analysis of downstream targets (*e.g.*, *HMOX1, SOD1, CAT, GSR, GSTA1, PRDX2)* of Nrf2 signaling with total RNAs isolated from the wound regions at both endpoints (d9/d20). (**g**) Nrf2, HO-1, Cat, Sod1, and β-actin protein expression levels were quantified using WES analysis on d9 (upper panel) and d20 (lower panel). Scale bar is 50 µm. Data are presented mean ± S.D.; *p < 0.05, **p < 0.01, ***p < 0.001, as related to control values (ctrl).

Cellular events such as mechanical and cytoskeletal input signaling, cell–cell interactions, and oxidative stress response pathways [16] are central mechanisms to control YAP/TAZ activity (schematic overview in Fig. 5). First, we quantified several targets of tight (tj) and adherence junctions (aj) by qPCR analysis. The mRNA levels of tj protein occludin (*OCLN*) were similar to those found in untreated wounds. In contrast, claudin 1 (*CLDN1*), the zonula occludens protein 1 (*ZO1*), and the aj associated β-catenin (*CTNNB1*) were upregulated mainly on d9. The aj-associated E-cadherin (*CDH1*) was significantly upregulated on d20 showing the differential regulation of gas plasma-induced treatment in dependence on wound healing stages (Fig. 5**a**). Protein levels of β-catenin and cadherin were investigated and tended to have expression levels similar to that of the mRNA (Fig. 5**b**). To show the gas plasma-stimulated differential expression and localization of aj proteins, we isolated skin cells from gas plasma-treated and untreated diabetic skin tissues. Again, representative images showed the gas plasma-induced change of localization and expression of β-catenin and E-cadherin (Fig. 5**c**).

Next, we investigated several transcription factors, such as the forkhead box O (FoxO) and nuclear factor erythroid 2-related factor 2 (Nrf2). The activation of FoxO proteins in response to oxidative stress is tightly regulated and integrated with other signaling pathways involved in cellular stress responses and the antioxidant defense system. Credence for this proposition is reflected by our data depicting antioxidant responses. We identified an upregulation of *FoxO* levels on d9 in contrast to d20 and to untreated wounds (Fig. 5**d**). Nrf2 regulates the expression of antioxidative response (ARE) genes. We found an upregulation of the master regulator of antioxidant defense *NRF2* as shown by qPCR (Fig. 5**d**) and by nuclear staining in gas plasma-treated wound tissue, particularly in cells of basal skin layers on d9 (Fig. 5**e**). Expectedly, a concomitant substantial increase of heme oxygenase 1 (*HMOX1*, gene transcript of HO-1 protein), cytoplasmic superoxide dismutase (*SOD1*), catalase (*CAT*), glutathione reductase (*GSR*), and glutathion (*GSTA1*) levels were detected along with an early (d9) and sustained (day 20) activation at both time points (Fig. 5**f**). However, the expression levels of NAD(P)H quinone oxidoreductase 1 (*NQO1*), Kelch- (*KEAP1*), peroxiredoxin 2 (*PRDX2*), and *SOD2* were similar to that of controls (data not shown). WES analysis on d9 (upper panel) and d20 (lower panel) confirmed a regulation of Nrf2, HO-1, Cat, and Sod1 proteins that were significantly increased after gas plasma treatment (Fig. 5**g**). Similar findings regarding expression levels were made in female and male mice (data not shown). In summary, gas plasma-mediated responses highlight the crosstalk and coordination between Nrf2 and FoxO in cellular stress responses, which share some common target genes involved in antioxidant defense.

## Discussion

The prevalence of diabetes mellitus is expected to increase in the coming years, largely due to a population that is aging and rising rates of obesity and sedentary lifestyles [54]. It is worth noting that diabetes is a chronic condition requiring high-cost treatments, and it can have serious complications if left untreated. Due to poor blood circulation, neuropathy, and hyperglycemia, non-healing wounds are one consequence in patients with diabetes [55]. The hampered healing process is characterized by a chronification of the inflammation, disrupted epithelialization and angiogenesis, as well as an imbalance in extracellular matrix regulation [55]. Although most animal models resembling human diabetes have limitations, the diabetic-inducing substance streptozotocin (STZ) is an established method to be injected into rodents [11]. Here, to represent the diversity seen in human diabetic patients, we have chosen this model to investigate impaired wound healing mechanisms. A multitude of preclinical [42], [45], [56], [57] and clinical studies suggest that medical gas plasma may be a promising approach for promoting wound healing [21], [58], [59], [60] and therapy of chronic inflammatory skin diseases [61], [62], [63]. However, the healing in diabetic wounds and especially the signaling pathways involved in the gas plasma-induced healing process are underexplored, and the current study aimed to address this gap.

Redox control, which refers to the regulation of cellular redox balance, is intricately involved in different stages of cellular and molecular events in wound healing aimed at repairing damaged tissue [18]. During wound formation, the initial injury triggers several cellular responses, including ROS production at the wound site. ROS serve as signaling molecules that regulate various aspects of wound healing, including inflammation, cell migration, proliferation, and tissue remodeling [64]. However, an excessive accumulation of ROS can lead to oxidative stress, which can impair wound healing by damaging cellular components and delaying tissue repair [65]. Therefore, proper regulation of ROS production, signaling, and antioxidant defense is essential to maintain the balance between pro-oxidant and antioxidant processes, ensuring an optimal wound healing environment and efficient tissue repair. Medical gas plasmas generate a multitude of ROS in the gas phase, which are transported directly to the defect tissue areas [66]. Due to these characteristics, gas plasma has been successfully used in wound closure studies (reviewed in [20]), affirming our findings of shortened wound closure together with an increased re-epithelialization of diabetic wounds. Similarly to gas plasma, topical ozone gas therapy was successfully used for infected dermal wounds due to the stimulating wound healing as well as the antimicrobial nature [67].

Under physiological conditions, the Hippo signaling pathway is a well-conserved signaling cascade that has a central role in regulating tissue growth, cell proliferation, homeostasis, and regeneration [68]. In recent years, there has been growing interest in the evaluation of the role of downstream effectors of Hippo signaling YAP/TAZ in regulating the behavior of various skin cell types involved in the repair process, including fibroblasts [69], keratinocytes [50], and immune cells [70]. Investigating gas plasma-induced effects in diabetic wounds, we found an increase in YAP expression, which was widely expressed in skin cells nine days after wounding. YAP entered the nucleus in damaged areas, suggesting activation of YAP signaling associated with an upstream-suppressed Hippo signaling as recently described [46]. The suppression of the Hippo pathway has also been demonstrated in epidermal keratinocytes to promote migration and proliferation, facilitating wound closure [50]. In contrast, activation of the Hippo pathway in dermal fibroblasts has been shown to inhibit their proliferation and migration, which can contribute to impaired wound healing [14]. *In vivo*, a skin-specific deletion of YAP in adult mice severely impairs regeneration after wounding [11], [71], [72], validating the important role of YAP signaling in wound closure. Additionally, the Hippo pathway is regulated by a series of signaling proteins and upstream regulators, such as kinases, phosphatases, cellular stress sensors, cell-junctional proteins, and mechanical stimuli that ultimately restrict the activity of YAP and its paralog transcriptional co-activator with PDZ-binding motif (TAZ) [73]. YAP/TAZ activity is essential in skin homeostasis as deletion of YAP/TAZ slows proliferation of basal layer cells and leads to hair loss and tissue damage [74]. After gas plasma treatment, TAZ was similarly expressed and located in the nuclei of some cells in the epidermis on d9 but overall to a lesser extent as YAP. The nuclear translocation of YAP and TAZ and their coactivation of TEAD (transcriptional activator domain) promoted the expression of cell-proliferative or antiapoptotic genes [75]. This ultimately leads to the activation of cell proliferation and survival necessary for tissue repair. The connective tissue growth factor (CTGF) and cysteine-rich angiogenic inducer 61 (Cyr61) were identified as YAP/TEAD target genes participating in their growth-supporting function [76]. Although CTGF shows low basal expression in normal skin, it is transiently upregulated after dermal injury [46]. We identified activation of TEAD downstream targets in gas plasma-treated wounds with increased expression of CTGF, Cyr61, and fibroblast growth factor 1 (FGF1) on d9, which is in accordance with an *in vitro* study in gas plasma-treated co-cultured skin cells [50]. Overall, elevated YAP/TAZ expression and nuclear localization were found, underlining the importance of YAP/TAZ signaling in gas plasma-induced diabetic wound healing.

Although the Hippo pathway controls YAP through serine or tyrosine phosphorylation [76] that promotes its sequestration and degradation, another important finding of our study was the only transiently gas plasma-induced transcriptional activation of YAP/TAZ. We have provided evidence of Hippo signaling activation in later wound healing stages (d20) as demonstrated by a strong expression and staining of phosphorylated (p)YAP in epidermal cells of healed regions. Thus, Hippo pathway activation prevented the nucleic localization and transcriptional activity of YAP/TAZ and promoted their phosphorylation and cytoplasmic retention, as shown 20 days after injury in the healed region. This finding is coherent with decreased expression levels of growth factors after gas plasma treatment. As shown in several cancer studies, overexpression of YAP causes suppression of apoptosis, growth factor-independent proliferation, and epithelial-to-mesenchymal transition (EMT). It has been, therefore, implicated as an oncogene in human tumors [12], [16], [76]. Moreover, our findings suggest that the Hippo pathway affects wound closure transiently through modulated expression and localization of YAP/TAZ observed in diabetic skin wounds in dependence on wound healing stages.

The molecular basis of the relationship between YAP/TAZ and downstream targets, which has also been implicated in diabetic disorders, was further depicted. Tumor necrosis factor (TGF) β is a multifunctional cytokine that plays an essential role during many stages of wound healing and is produced by various cell types, including platelets, macrophages, and fibroblasts [77]. TGFβ1 is reduced in the wound fluid of diabetic patients compared to non-diabetic patients [78], which could add to delayed wound healing in diabetes. Furthermore, hyperglycemia, a hallmark of diabetes, can impair TGFβ signaling by increasing the production of advanced glycation end products (AGEs). AGEs are generated if glucose reacts with proteins in the body and can interfere with TGFβ signaling by binding to its receptors and inhibiting its activity [79]. Additionally, YAP/TAZ-deficient fibroblasts are less reactive to TGFβ1 stimulation *in vitro*, produce less extracellular matrix (ECM) components, have lower expression of myofibroblast markers such as smooth-muscle actin alpha (αSMA), and show lower contractile capabilities [69], suggesting a link between activation of YAP/TAZ and the production of TGFβ1 [46]. TGFβ1 is a major regulator of collagen biosynthesis [80]. It is involved in subsequent wound contraction [81] and vascular smooth muscle cell differentiation [82] during wound healing [83]. Gas plasma-mediated increase of collagen fibers in re-epithelialized regions leading to the formation of granulation tissue underlines the finding of enhanced ECM production and proliferation of fibroblasts in our diabetic model, which was also demonstrated in other studies [46]. Additionally, CTGF enhanced the transforming growth factor (TGF) β-induced phosphorylation and nuclear translocation of Smad2/3. Smad2 is intimately involved in wound healing and depends on TGFβ signaling [84]. Although some studies have shown that YAP interacts with TGFβ signaling [85], other reports provided evidence that TGFβ signaling is independent of the Hippo signaling pathway [46], [69]. Nevertheless, in diabetic wounds, TGFβ1 levels were increased in the dermis following gas plasma treatment, particularly in earlier wound healing stages.

Altered mechanotransduction or signaling through TGFβ or β-catenin pathways likely promotes aberrant YAP/TAZ activity in different contexts [14]. Plasma treatment with microwave plasma stimulates dermal papilla cell proliferation by activating YAP/TAZ and β-catenin signaling [86]. Several studies have shown that TGFβ1, YAP, and β-catenin can interact with each other to regulate various cellular processes involved in wound healing. As mentioned above, TGFβ1 has been shown to regulate the activity of YAP and β-catenin in many types of cells, including epithelial cells and fibroblasts. β-catenin is a protein involved in the canonical Wnt signaling pathway, which regulates differentiation, cell proliferation, and survival. In the absence of Wnt ligands, β-catenin is phosphorylated and targeted for degradation by a destruction complex. In the presence of Wnt ligands, the destruction complex is inhibited, leading to the accumulation of β-catenin in the cytoplasm and its translocation to the nucleus, where it activates target genes involved in cell fate determination and tissue regeneration [87]. YAP and β-catenin have been shown to interact with each other to co-activate target genes and regulate cell proliferation, differentiation, and tissue regeneration during wound healing [88]. Moreover, the crosstalk between YAP and β-catenin signaling can modulate the expression of genes involved in EMT, an important process in wound healing and tissue repair. Ectopic expression of an activated YAP mutant or dysregulation of upstream regulators of YAP localization − such as the cell adhesion regulator α-catenin − results in the expansion of epidermal basal cells, which resembles an uncontrolled epidermal damage response. Moreover, TGFβ1 can activate β-catenin signaling, leading to the expression of genes involved in ECM synthesis and tissue remodeling [77]. In this regard, further studies of gas plasma effects are indispensable.

As mentioned above, redox control mechanisms are essential for maintaining the delicate balance between ROS production and their detoxification during wound healing. First, inflammatory cells, such as neutrophils and macrophages, are involved in the early stages of wound healing. These cells generate ROS as part of their antimicrobial defense mechanism [89]. Redox signaling through ROS regulates the inflammatory response, including immune cell recruitment and release of cytokines and chemokines. Recent studies have also suggested that the Hippo pathway may regulate the immune response in wound healing. For example, activation of the Hippo pathway in macrophages has been shown to promote their polarization towards an anti-inflammatory M2 phenotype, which can promote tissue repair and reduce inflammation. In contrast, inhibition of the Hippo pathway in macrophages can lead to polarization towards a pro-inflammatory M1 phenotype, which can impede wound healing [90]. Additionally, TGFβ1 is involved in the recruitment and activation of immune cells, which are indispensable for the clearance of debris and bacteria from the wound site [91].

Redox-sensitive signaling pathways, such as nuclear factor Nrf2, are modulated to regulate gene expression and cell behavior during wound healing [92]. The antioxidant defense system plays a decisive role in maintaining redox balance and promoting optimal wound healing. A dysregulation in Nrf2 signaling is strongly associated with a diabetic phenotype [93], [94]. Nrf2 levels in total cell extracts of diabetic wounds were diminished, and this abnormality appears to stem from a diabetes-related decrease in Nrf2 protein stability [94]. Following gas plasma treatment, we could demonstrate activation of Nrf2 with its nuclear translocation *ex vivo* in dermal fibroblasts and epidermal keratinocytes isolated from diabetic mouse skin as well as *in vivo* in diabetic wound sites. This was associated with an increase in the antioxidant capacity and a decrease in the sensitivity of skin cells to gas plasma-induced apoptosis, as shown previously in our studies [45], [95], [96]. Additionally, crosstalk and coordination exist between Nrf2 and another transcription factor, FoxO, in cellular stress responses to amplify the cellular response to oxidative stress [97]. Studies have indicated that Nrf2 [98] and FoxO [99] can co-bind specific DNA sequences and synergistically share the expression of some common targets involved in antioxidant response. This includes enzymatic antioxidants such as superoxide dismutase, catalase, and glutathione peroxidase, scavenging excess ROS, maintaining redox homeostasis during the inflammatory phase, preventing oxidative damage, and aiding in wound healing. Moreover, FoxO and YAP can interact with each other to regulate various cellular processes and downstream effectors, including cell proliferation, differentiation, and survival [100]. The balance between FoxO and YAP activity is critical for maintaining tissue homeostasis and averting disease development such as cancer and fibrosis. FoxO and YAP can also interact with common downstream effectors to regulate cell fate decisions. For example, both FoxO and YAP can regulate the cell cycle inhibitor p21 expression, which is key for regulating differentiation and cell proliferation, and genes involved in epithelial-mesenchymal transition (EMT), a process involved in wound healing and tissue repair [101].

Redox signaling is crucial for the migration and proliferation of cells involved in wound healing, where ROS acts as secondary messengers. Besides TGFβ1 [102], angiomotin (AMOT) plays a multifaceted role in angiogenesis, new blood vessel formation that supplies nutrients and oxygen to healing tissue during embryogenesis, and pathological angiogenesis [103]. It acts as a regulator of endothelial cell migration by interacting with various signaling pathways and proteins involved in cell motility, such as the Rho GTPases and the Hippo signaling pathway [104]. AMOT is involved in proper vascular development, modulating VEGF receptor signaling and downstream pathways, contributing to endothelial cell–cell adhesion and barrier integrity. It interacts with proteins involved in endothelial barrier function, including the tight junction protein zonula occludens-1 (ZO-1). Hence, AMOT contributes to the maintenance of vascular integrity [105], underlining the importance of gas plasma-induced alterations of AMOT (Fig. S1**c**) and ZO-1 in diabetic wounds.

Our study had several limitations, *e.g.*, our inability to correlate the transcriptomic data with proteomic data due to the lack of sufficient wound tissue. In addition, we are aware that the chip-based transcriptomic microarray technique used in this study may not be comparable to modern RNA-sequencing approaches. Moreover, due to ethical constraints, we were able to conclude the findings in only one but not more mouse strains.

## Conclusion

YAP/TAZ, TGFβ, and β-catenin are three signaling pathways affected in gas plasma-treated wounds in our diabetic mouse model. They engaged in mutual interaction to oversee diverse cellular functions crucial for wound healing, such as cell growth and extracellular matrix synthesis. Additionally, our research emphasized that repetitive *in vivo* application of reactive species derived from gas plasma facilitated wound closure in a diabetic mouse model. The gas plasma-induced regulation of the Hippo pathway presents a highly promising outcome for employing plasma technology in diabetic wound healing. Both emerging evidence and our study indicate that the Hippo signaling significantly influences multiple facets of the repair process. Further research is needed to study the effects of other clinically relevant co-morbidities, such as immune suppression and aging, in relation to gas plasma-induced wound healing and its mechanisms.

## Declaration of Competing Interest

The authors declare that they have no known competing financial interests or personal relationships that could have appeared to influence the work reported in this paper.
