# Supplementary material for: YAP/TAZ, beta-catenin, and TGFb pathway activation in medical plasma-induced wound healing in diabetic mice
Source: J Adv Res. 2024 Jul 8;72:387–400. doi: 10.1016/j.jare.2024.07.004 (PMC12147638; doi:10.1016/j.jare.2024.07.004)
Supplement: Supplementary Data 1 [file mmc1.docx]

**Supplemental data**

Table S1. Murine gene-specific primers used in qPCR.

| **Gene name** | **Gene ID** | **Primer sequences (3`- 5`)** |
| --- | --- | --- |
| yes-associated protein 1 | *YAP* | TGA GAT CCC TGA TGA TGT ACC AC  TGT TGT TGT CTG ATC GTT GTG AT |
| transcriptional coactivator with PDZ-binding motif | *TAZ* | ATGCCCCTCCATGTGAAGTG  GTGCCAACTAGGCCCATGAC |
| neurofibromatosis type II | *NF2* | GGCCAAGTATGGCGACTATGA  ATCACCCTTTTCGGGAGCAAT |
| sirtuin 1 | *SIRT* | TGATTGGCACCGATCCTCG  CCACAGCGTCATATCATCCAG |
| macrophage stimulating 1 | *MST1* | TGGTGCTACACAACAAACCG  CCTCACCGTTGCACAGAACA |
| salvador homolog 1 | *SAV1* | CTGTCCCGCAAGAAAACCAAA  AATGAAGGCATGAGATTCCGC |
| large tumor suppressor kinase 1 | *LATS1* | AAAGCCAGAAGGGTACAGACA  CCTCAGGGATTCTCGGATCTC |
| MOB kinase activator 1A | *MOB1* | ATGAGCTTCTTGTTTGGTAGTCG  ATGACAGCCATCCGTAGGTTG |
| transcriptional enhancer factor TEF-1 | *TEAD1* | GAGCGACTCGGCAGATAAGC  CCACACGGCGGATAGATAGC |
| nuclear factor erythroid 2-related factor 2 | *NRF2* | GAG TCG CTT GCC CTG GAT ATC  TCA TGG CTG CCT CCA GAG AA |
| heme oxygenase 1 | *HMOX1* | TGA AGC AGG CAT CTG AGG G  CGA AGG TGG AAG AGT GGG AG |
| catalase | *CAT* | CAG AGA GCG GAT TCC TGA GAG A  CTT TGC CTT GGA GTA TCT GGT GAT |
| superoxide dismutase [Cu-Zn] | *SOD1* | GAA ACA AGA TGA CTT GGG CAA AG  TTA CTG CGC AAT CCC AAT CA |
| glutathione-disulfide reductase | *GSR* | TCG GAA TTC ATG CAC GAT CA  GGC TCA CAT AGG CAT CCC TTT |
| glutathione S-transferase A1 i | *GSTA1* | CAG CCT GGC AGC CAG AGA  TCT GTG GCT CCA TCA ATG CA |
| peroxiredoxin 2 | *PRDX2* | GGT GCC TTC AAG GAA ATC AA  GCC TAG CTT TCG GAA GTC CT |
| colony-stimulating growth factor | *CTGF (CSF2)* | GAC CCA ACT ATG ATG CGA GCC  CCC ATC CCA CAG GTC TTA GAA C |
| cysteine-rich angiogenic inducer 61 | *CYR61* | TAA GGT CTG CGC TAA ACA ACT C  CAG ATC CCT TTC AGA GCG GT |
| fibroblast growth factor 1 | *FGF1* | CAGCTCAGTGCGGAAAGTG  ATAAAAGCCCTTCGGTGTCCA |
| amphiregulin | *AREG* | GGGGACTACGACTACTCAGAG  TCTTGGGCTTAATCACCTGTTC |
| jagged 1 | *JAG1* | AATCGCATCGTACTGCCTTTC  GTGTCATTACTGGAATCCCAGG |
| tumor necrosis factor beta 1 | *TGFB1* | TTT GGA GCC TGG ACA CAC AGT ACA  TGT GTT GGT TGT AGA GGG CAA GGA |
| mothers against decapentaplegic homolog 2 | *SMAD2* | TCCGTACCACTACCAGAGAGT  GGCGGCAGTTCTGTTAGAATC |
| interleukin 1β | *IL1β* | GCA ACT GTT CCT GAA CTC AAC T  ATC TTT TGG GGT CCG TCA ACT |
| cyclin-dependent kinase inhibitor 1 | *CDKN1A* | GGA ATT GGA GTC AGG CGC AGA T  GAA GAG ACA ACG GCA CAC TTT GCT |
| β-catenin | *CTNNB1* | CCC AGT CCT TCA CGC AAG AG  CAT CTA GCG TCT CAG GGA ACA |
| E-cadherin | *CDH1* | CAC CTG GAG AGA GGC CAT GT  TGG GAA ACA TGA GCA GCT CT |
| occludin | *OCLN* | TGT GGG ATA AGG AAC ACA TTT ATG A  CAG ACA CAT TTT TAA CCC ACT CTT CA |
| claudin 1 | *CLDN1* | GGG GAC AAC ATC GTG ACC G  AGG AGT CGA AGA CTT TGC ACT |
| zona-occludens protein 1 | *ZO-1* | TGA ACG CTC TCA TAA GCT TCG TAA  ACC GTA CCA ACC ATC ATT CAT TG |
| angiomotin | *AMOT* | CCG CCA GAA TACC CTT TCA AG  GGT TCA GGC GAT GCT CAC TA |
| angiomotin like 1 | *AMOTL1* | CCT TGC GAG CCT GTG CTT A  AAG TCT GGG TAG AAG TAG GCG |
| angiomotin like 2 | *AMOTL2* | GTA TTT GGA AGA ACG GGC TAT GA  CCG GAT GAG AGT GGT ATC GC |
| β actin | *ACTB* | TTG CTG ACA GGA TGC AGA AG  ACA TCT GCT GGA AGG TGG AC |
| glyceraldehyde 3-phosphate dehydrogenase | *GAPDH* | CAT GGC CTC CAA GGA GTA AG  TGT GAG GGA GAT GCT CAG TG |
| ribosomal protein 13A | *RLP13A* | AGC CTA CCA GAA AGT TTG CTT AC  GCT TCT TCT TCC GAT AGT GCA TC |

*Figure S1*


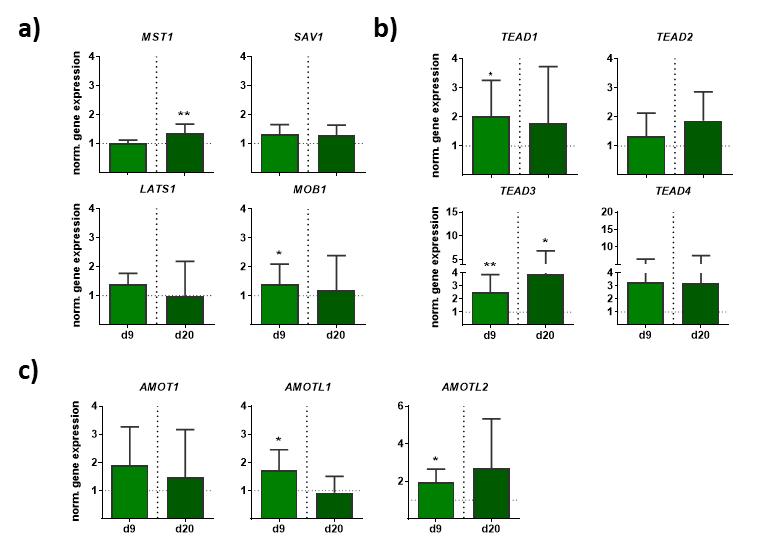


Figure S1. **Gas plasma treatment modulated the Hippo signaling pathway in murine diabetic wounds.** Ear wounds of mice were treated with gas plasma as described. (**a**) Gene expression levels of upstream kinases (*e.g.*, *MST1, SAV1, LATS1, MOB1*), (**b**) the transcription factor TEA domain family (*TEAD1-4*), and (**c**) angiomotin (*AMOT1* and *AMOTL1/2*) on both endpoints. Data are presented as mean ±S.D.; *p<0.05, **p<0.01, ***p< 0.001, as compared to controls (ctrl).
